# Chlamydia trachomatis and mycoplasma infections in tubal pregnancy

**DOI:** 10.1038/s41598-019-52193-7

**Published:** 2019-11-04

**Authors:** Yang Liu, Yunjiang Zhang, Dehong Yang, Changjun Xu, Yajuan Huang, Qing Qing, Daizhu Li, Jing Liao, Yulu Ding, Jiaoyue Zhou, Jie Zhang, Chunyi Sun, Honglin Zhou

**Affiliations:** 1 Department of Reproductive, The Second Affiliated Hospital of Kunming Medical University, Kunming, Yunnan 650101 P.R. China; 2 Department of Reproductive, The Second People’s Hospital of Yunnan Province, Kunming, Yunnan 650101 P.R. China; 3 Department of the reproductive gynecology, The First People’s Hospital of Yunnan Province, Kunming, Yunnan 650101 P.R. China; 4 Department of Gynecology, The Second Affiliated Hospital of Kunming Medical University, Kunming, Yunnan 650101 P.R. China; 5 Department of Surgery, The Second Affiliated Hospital of Kunming Medical University, Kunming, Yunnan 650101 P.R. China

**Keywords:** Drug discovery, Medical research

## Abstract

*Chlamydia trachomatis* (CT) infection is an important factor for tubal pregnancy. However, whether *Ureaplasma urealyticum* (UU) and *Mycoplasma hominis* (MH) infections are also involved in tubal pregnancy remains unknown. This study is aimed to detect CT, UU, and MH in cervical secretions from patients with tubal pregnancy and control women in early pregnancy, to explore their prevalence rates and drug susceptibilities. Analysis was performed on patients with tubal pregnancy and those requiring termination of early pregnancy at <12 weeks from July 2013 to March 2014. Cervical secretions were tested for UU/MH with a UU/MH isolation and culture kit and for CT antigen by an immunochromatographic assay. Mycoplasma samples were tested for resistance to 12 antibiotics. There were no cases of CT infection detected. Mycoplasma infection rates (single or mixed) were similar in the tubal pregnancy and control groups, but the total rate of infection was higher for tubal pregnancy. All MH samples were sensitive to tetracyclines as well as josamycin and azithromycin. Josamycin and clarithromycin were effective against all UU cultures. Over 50% of the samples tested were resistant to ciprofloxacin.

## Introduction

The implantation of fertilized eggs outside the uterine cavity is referred to as ectopic pregnancy, with a morbidity of about 2%^1^. Tubal pregnancy is the most common type of ectopic pregnancy in women of childbearing age, accounting for about 95% of all cases^2^. Some ectopic pregnancies resolve spontaneously. Because the fallopian tubes are not conducive to placental implantation and embryonic development, the risk of tubal pregnancy rupture and bleeding is enormous^3^. The risk of ectopic pregnancy is increased in women with histories of ectopic pregnancy, infection, infertility, adnexal surgery, appendectomy, and the use of intrauterine devices^4^.

Salpingitis, inflammation of the fallopian tubes, caused by pathogenic microorganisms is a major cause of tubal pregnancy^1^. Previously, bacteria and *Neisseria gonorrhoeae* were considered the main pathogens in salpingitis^5^. With the progress of medicine and the improvement of detection methods, *Chlamydia trachomatis* (CT) has been implicated as a leading cause of salpingitis, ectopic pregnancy, and infertility^6^. *Ureaplasma urealyticum* (UU) and *Mycoplasma hominis* (MH) are often found alongside CT^7^. However, the genital tract flora of sexually active healthy women often includes MH and UU^8^. Therefore, it is not entirely clear whether UU and MH are also implicated in salpingitis or tubal pregnancy^9^. Meanwhile, MH and UU are implicated in pelvic inflammatory disease, which is related to tubal pregnancy^10^. In addition, both are also involved in adverse pregnancy outcomes^11^. However, a study suggested that there are no associations of MH and UU serum antibodies with ectopic pregnancy^12^.

Whether treatment for MH and UU is required remains controversial, but in some pregnant women, it might be important because co-infection with UU and MH could increase the odds of preterm delivery^13^ and low birth weight infants^14^. Antibiotic treatment for MH and UU involves drugs that interfere with protein synthesis and inhibit topoisomerases^8^. However, previous studies have suggested that there is a high degree of antibiotic resistance in samples of MH and UU^8,10,15^. Therefore, it is important to monitor drug resistance for isolated UU and MH samples to ensure that effective treatment is provided to resolve infection, where it is considered appropriate.

The aim of this study was to detect CT, UU, and MH in the cervical secretions of patients with tubal pregnancy and women in early pregnancy, exploring their prevalence rates, drug susceptibilities, and relationships with tubal pregnancy.

## Results

### Baseline characteristics

The baseline characteristics of the two groups are shown in Table 1. There were 81 women included in the tubal pregnancy group and 102 in the control group. The mean age and gestational age were similar in both groups.

**Table 1:** Baseline characteristics of the two patient groups.

	Tubal pregnancy group n = 81	Control group n = 102
Age, years (mean ± SD)	29 ± 2.6	30 ± 1.8
Gestational age, weeks (mean ± SD)	25.0 ± 2.8	26.0 ± 2.1
**Parity history (n**, **%)**		
0	50 (61.7%)	31 (30.4%)
1	23 (28.4%)	48 (47.1%)
≥2	8 (9.9%)	23 (22.5%)
**History of abortion (n**, **%)**		
0	18 (22.2%)	42 (41.2%)
1	41 (50.6%)	37 (36.3%)
≥2	22 (27.2%)	23 (22.5%)
PID history (n, %)	0	0

PID = pelvic inflammatory disease.

### CT, MH and UU infection rates

No CT infection cases were detected in any of the cervical samples. The rates of MH, UU, and MH + UU infections are shown in Table 2. There were no significant differences between the two groups in terms of MH, UU, or MH + UU infection rates. However, when the overall mycoplasma infection rates were compared, the tubal pregnancy group showed a significantly higher value (66.7% vs. 49.0%, P = 0.017).

**Table 2:** Detection of chlamydia and mycoplasma in cervical secretions.

	Tubal pregnancy group n = 81	Control group n = 102	P value
CT	0	0	—
MH only (n, %)	6 (7.4%)	2 (2.0%)	0.141
UU only (n, %)	45 (55.6%)	46 (45.1%)	0.160
MH + UU (n, %)	3 (3.7%)	2 (2.0%)	0.656
Total (n, %)	54 (66.7%)	50 (49.0%)	0.017

CT = *Chlamydia trachomatis;* UU = *Ureaplasma urealyticum;* MH = *Mycoplasma hominis*.

### Antibiotic resistance of the mycoplasma samples

The MH and UU samples were tested for resistance to 12 antibiotics, including tetracyclines (tetracycline, doxycycline, and minocycline), macrolides (josamycin, erythromycin, roxithromycin, azithromycin, and clarithromycin), and quinolones (levofloxacin, ciprofloxacin, ofloxacin, and sparfloxacin) as shown in Table 3. The results showed that all MH samples were sensitive to the three tetracyclines tested, and the macrolides josamycin and azithromycin. However, of these drugs only josamycin was effective against all UU samples. The highest level of resistance was found to ciprofloxacin, with over 50% of all the samples tested being resistant to this drug.

**Table 3:** Drug susceptibility findings.

Drug resistance	Tubal pregnancy group			Control group		
	MH only (n = 6)	UU only (n = 45)	MH + UU (n = 3)	MH only (n = 2)	UU only (n = 46)	MH + UU (n = 2)
Tetracycline	0	3 (6.7%)	1 (33.3%)	0	4 (8.7%)	1 (50.0%)
Doxycycline	0	1 (2.2%)	0	0	1 (2.2%)	0
Minocycline	0	0	0	0	1 (2.2%)	0
Josamycin	0	0	0	0	0	0
Erythromycin	1 (16.7%)	5 (11.1%)	1 (33.3%)	0	4 (8.7%)	1 (50.0%)
Roxithromycin	2 (33.3%)	2 (4.4%)	0	1 (50.0%)	0	1 (50.0%)
Azithromycin	0	0	0	0	1 (2.2%)	0
Clarithromycin	2 (33.3%)	0	1 (33.3%)	0	0	1 (50.0%)
Levofloxacin	1 (16.7%)	3 (6.7%)	1 (33.3%)	0	8 (17.4%)	0
Ciprofloxacin	4 (66.7%)	26 (57.8%)	2 (66.7%)	2 (100.0%)	28 (60.9%)	2 (100.0%)
Ofloxacin	0	4 (8.9%)	1 (33.3%)	1 (50.0%)	8 (17.4%)	1 (50.0%)
Sparfloxacin	0	18 (40.0%)	3 (100.0%)	1 (50.0%)	22 (47.8%)	2 (100.0%)

UU = *Ureaplasma urealyticum;* MH = *Mycoplasma hominis*.

## Discussion

The aim of this study was to detect CT, UU, and MH in the cervical secretions of patients with tubal pregnancy and compare their rates to those of control women in early pregnancy. These findings would provide insights into infection prevalence and drug susceptibilities of the involved infectious agents, and may help establish a potential relationship between infection and tubal pregnancy. As shown above, there were no cases of CT infection, and UU, MH, and MH + UU infection rates were similar in the tubal pregnancy and control groups. However, the total rate of UU and MH infection was higher in tubal pregnancy. All MH samples were sensitive to tetracyclines as well as josamycin and azithromycin. Josamycin and clarithromycin were effective against all UU cultures. Over 50% of all the samples tested were resistant to ciprofloxacin.

Many previous studies have shown a link between CT infection and tubal pregnancy^16–18^. However, few studies have investigated the roles of UU and MH. The above results showed no overt associations of UU and MH infection with tubal pregnancy as the infection rates were similar in both patient groups. This supports the conclusions of a previous study^12^. However, when the total rates of UU and MH infections were compared, there was a significant difference between the two groups. This suggests that larger studies are needed to fully investigate whether UU and MH infections play a role in tubal pregnancy.

In this study, CT was not detected, but this might be due to the low sensitivity of the applied detection method. Cell culture is a more sensitive and specific method for the diagnosis of reproductive tract CT, but it has high requirements for laboratory equipment and operation and takes a long time to produce results; therefore, it is usually not used as a routine clinical examination and for epidemiological screening, but as a reference standard for other methods. The immunochromatographic method in this study employed a commercial kit that is simple to operate and easily generates immediate results, which is suitable for outpatient examination. Its specificity is 100%, for a sensitivity of 75–85% based on cell culture methods according to the manufacturer. Nucleic acid amplification diagnosis is available for routine clinical use for CT, UU and MH infection detection, and has higher sensitivity, but is more expensive and therefore cost-prohibitive in many clinics in China. In addition, the results are also not immediately available. As less expensive polymerase chain reaction (PCR) protocols for CT, MH, and UU detection have been developed, sensitivity in future studies might improve^19^.

There is a global variation in the sensitivities of UU and MH samples to different antibiotics^10^. The sensitivity and susceptibility of mycoplasma to antibiotics are also not static^20^. A Serbian study found doxycycline to be the most effective drug^8^ while a Chinese report suggested that josamycin, doxycycline, and minocycline are all effective^15^. Meanwhile, a German study found clarithromycin and josamycin are the most potent macrolides, although doxycycline was considered the first-choice for the treatment of UU infections and may be used for co-infection with MH^20^. In Greek women with clinical vaginitis, MH isolates were 100% susceptible to tetracycline and doxycycline, and tetracycline and doxycycline were also the most effective drugs for UU although some resistant cases were detected^21^. This study showed similar results in that the susceptibilities of the various mycoplasma infections were different. All three tetracyclines and azithromycin were effective against all MH cultures, and clarithromycin was effective against UU; only josamycin was effective against both UU and MH. The discrepant results of these studies emphasize that it is important for drugs to be selected according to drug susceptibility test data, with rational treatment performed to improve the clinical efficacy and reduce the occurrence of drug resistance.

An important point has to be considered. *M*. *hominis* is intrinsically resistant to erythromycin and 14- and 15-membered macrolides because of a SNP conferring macrolide resistance in the 23S rRNA gene^22–24^. Surprisingly, in the present study, all *M*. *hominis* strains were sensitive to azithromycin and to some 14- and 15-membered macrolides. These results highlight the intrinsic uncertainty of drug susceptibility tests. Unfortunately, the strains were not available for retest or for other tests for all patients. A loss of mutation or novel mutations in 23S rRNA conferring sensitivity to erythromycin and azithromycin are possible^25^. Sequencing should be performed in the future. Therefore, these results should not be used as treatment guide for clinicians and will require validation in the future.

This study had some limitations. The sample size was quite small, and a larger study in multiple centers might provide more convincing evidence of a difference in mycoplasma infection between the two groups. The retrospective nature of this study is another limitation; indeed, while we can provide data on infection prevalence, it is difficult to draw any conclusions on the roles of these infections in tubal pregnancy. The testing procedures for infection were not as sensitive as PCR based assays that are currently used in some clinics. This may be the reason for the lack of CT detection in this study. In addition, bacterial load was not precisely controlled in susceptibility tests, which may affect MIC readings. We would have tested for *Mycoplasma genitalium*, which has demonstrated association with tubal pregnancy. Finally, we did not assess the involvement of host parameters such as immune cell profiles and cytokine production, which deserves further attention.

Overall, no CT infection cases were detected in women with tubal pregnancy or normal early pregnancy. UU, MH, and UU + MH infection rates were similar in both groups, but the total rate of UU and MH infection was higher in women with tubal pregnancy. Therefore, further studies are required to establish the associations of UU and MH infections with tubal pregnancy. UU and MH samples showed some resistance to antibiotics, with the least effective treatment likely to be ciprofloxacin. However, josamycin was fully effective at preventing the growth of mycoplasma cultures in the present study.

## Methods

### Patients

A retrospective study was conducted. Patients admitted to the Gynecologic Outpatient Clinic and Gynecological Inpatient Department of Second Affiliated Hospital of Kunming Medical University from July 2013 to March 2014 were selected, and their cervical secretions were collected for mycoplasma culture and *Chlamydia trachomatis* detection.

This study was approved by the ethics committee of the above hospital, who waived the need for consent because of the retrospective nature of this study. All methods were performed in accordance with the relevant guidelines.

Patients were selected for inclusion in the tubal pregnancy group based on the following criteria: (1) tubal pregnancy, (2) admission to the Gynecological Inpatient Department during the study period, (3) investigation for CT and mycoplasma infections. The control group included individuals meeting the following criteria: (1) requiring termination of early pregnancy at <12 weeks; (2) reason for termination not due to an abnormality with the pregnancy, or health risk to the patient or fetus; (3) investigation for CT and mycoplasma infection; (4) treatment during the study period in the Gynecologic Outpatient Clinic. Exclusion criteria for both groups were: (1) a history of tubal pregnancy; (2) use of any antibiotics in the previous two weeks or use of vaginal irrigation within three days.

B ultrasound was used as the diagnostic method for intrauterine pregnancy, and postoperative pathological examination was the diagnostic technique for ectopic pregnancy.

### Data collection

The baseline data of the patients were collected from the hospital database. This included the patient’s age, gestational age, delivery time, history of abortion, history of pelvic inflammatory disease, and history of sexually transmitted diseases.

### Collection of cervical specimens

Sterilized cotton swabs were inserted 2–4 cm into the cervical orifice for sampling cervical secretions. Two senior-technicians analyzed the samples on the same day, in a blinded manner.

### Detection of UU and HM

Cervical samples were first grown in liquid cultures in an incubator at 35–37 °C. The samples were investigated for UU and MH with a UU/MH isolation and culture kit (Zhuhai Diel Bioengineering Co., Ltd, China). The media contained urea and arginine to allow the growth of UU and MH, respectively. In addition, the media changed color when urea and arginine were degraded into an alkaline substance because of the pH indicator phenolsulfonphthalein. After a 48-hour observation, yellow or orange-yellow color showed the sample was negative for UU or MH; clear transparent red indicated a positive result. A turbid red sample suggested contamination.

The kit also tested the resistance of UU and MH cultures to 12 antibiotics, including tetracycline, doxycycline, minocycline, josamycin, erythromycin, roxithromycin, azithromycin, clarithromycin, levofloxacin, ciprofloxacin, ofloxacin, and sparfloxacin. The highest drug concentration was in the upper row of wells and the lowest in the bottom row. A well-turning red indicated UU or MH growth; no color change suggested sensitivity to the drug. When only the lower row turned red, mycoplasma organisms were considered to be moderately sensitive; when both the upper and lower rows turned red, mycoplasma organisms were considered to be drug-resistant.

### CT detection

An immunochromatographic assay was used to detect CT antigen with the chlamydia trachomatis test kit (Unipath Ltd., UK). A positive test was reflected by a line appearing in the specimen window.

### Statistical analysis

Data are mean ± standard deviation (SD) or frequency and percentage and were analyzed with SPSS 22.0 (IBM Corp., NY, USA). Chi-squared or Fisher Exact tests were performed for between-group comparison, as appropriate. Two-sided P < 0.05 was considered statistically significant.

## Data Availability

The data set supporting the results of this report are included in the article.

## References

[1] Marion LL, Meeks GR (2012). Ectopic pregnancy: History, incidence, epidemiology, and risk factors. Clin Obstet Gynecol..

[2] Varma, R., Gupta, J. Tubal ectopic pregnancy. *BMJ Clin Evid* (2012). PMC3285146 22321966

[3] Hsu JY (2017). Disparities in the management of ectopic pregnancy. Am J Obstet Gynecol..

[4] Li C (2015). Risk factors for ectopic pregnancy: a multi-center case-control study. BMC Pregnancy Childbirth..

[5] Wasserheit JN (1986). Microbial causes of proven pelvic inflammatory disease and efficacy of clindamycin and tobramycin. Ann Intern Med..

[6] Bebear C, de Barbeyrac B (2009). Genital Chlamydia trachomatis infections. Clin Microbiol Infect..

[7] Guven MA, Dilek U, Pata O, Dilek S, Ciragil P (2007). Prevalance of Chlamydia trochomatis, Ureaplasma urealyticum and Mycoplasma hominis infections in the unexplained infertile women. Arch Gynecol Obstet..

[8] Skiljevic D, Mirkov D, Vukicevic J (2016). Prevalence and antibiotic susceptibility of Mycoplasma hominis and Ureaplasma urealyticum in genital samples collected over 6 years at a Serbian university hospital. Indian J Dermatol Venereol Leprol..

[9] Taylor-Robinson D, Jensen JS, Svenstrup H, Stacey CM (2012). Difficulties experienced in defining the microbial cause of pelvic inflammatory disease. Int J STD AIDS..

[10] Leli C (2012). Prevalence and antimicrobial susceptibility of Ureaplasma urealyticum and Mycoplasma hominis in a population of Italian and immigrant outpatients. Infez Med..

[11] Capoccia R, Greub G, Baud D (2013). Ureaplasma urealyticum, Mycoplasma hominis and adverse pregnancy outcomes. Curr Opin Infect Dis..

[12] Karaer A, Mert I, Cavkaytar S, Batioglu S (2013). Serological investigation of the role of selected sexually transmitted infections in the aetiology of ectopic pregnancy. Eur J Contracept Reprod Health Care..

[13] Lee MY, Kim MH, Lee WI, Kang SY, Jeon YL (2016). Prevalence and Antibiotic Susceptibility of Mycoplasma hominis and Ureaplasma urealyticum in Pregnant Women. Yonsei Med J..

[14] Bayraktar MR, Ozerol IH, Gucluer N, Celik O (2010). Prevalence and antibiotic susceptibility of Mycoplasma hominis and Ureaplasma urealyticum in pregnant women. Int J Infect Dis..

[15] Zeng XY, Xin N, Tong XN, Wang JY, Liu ZW (2016). Prevalence and antibiotic susceptibility of Ureaplasma urealyticum and Mycoplasma hominis in Xi’an, China. Eur J Clin Microbiol Infect Dis..

[16] Bakken IJ, Skjeldestad FE, Nordbo SA (2007). Chlamydia trachomatis infections increase the risk for ectopic pregnancy: a population-based, nested case-control study. Sex Transm Dis..

[17] Hillis SD, Owens LM, Marchbanks PA, Amsterdam LF, Mac Kenzie WR (1997). Recurrent chlamydial infections increase the risks of hospitalization for ectopic pregnancy and pelvic inflammatory disease. Am J Obstet Gynecol..

[18] Rekart ML (2013). Chlamydia public health programs and the epidemiology of pelvic inflammatory disease and ectopic pregnancy. J Infect Dis..

[19] Aguilera-Arreola MG, Gonzalez-Cardel AM, Tenorio AM, Curiel-Quesada E, Castro-Escarpulli G (2014). Highly specific and efficient primers for in-house multiplex PCR detection of Chlamydia trachomatis, Neisseria gonorrhoeae, Mycoplasma hominis and Ureaplasma urealyticum. BMC Res Notes..

[20] Krausse R, Schubert S (2010). *In-vitro* activities of tetracyclines, macrolides, fluoroquinolones and clindamycin against Mycoplasma hominis and Ureaplasma ssp. isolated in Germany over 20 years. Clin Microbiol Infect..

[21] Kechagia N, Bersimis S, Chatzipanagiotou S (2008). Incidence and antimicrobial susceptibilities of genital mycoplasmas in outpatient women with clinical vaginitis in Athens, Greece. J Antimicrob Chemother..

[22] Bebear, C. M. & Kempf, I. Antimicrobial therapy and antimicrobial resistance. In: Blanchard A, Browning G.F., editors. Mycoplasmas: Molecular Biology, Pathogenicity and Strategies for Control. Norfolk: Horizon Biosciences (2005).

[23] Pereyre S (2002). Mutations in 23S rRNA account for intrinsic resistance to macrolides in Mycoplasma hominis and Mycoplasma fermentans and for acquired resistance to macrolides in M. hominis. Antimicrob Agents Chemother..

[24] Valentine-King, M. A. & Brown, M. B. Antibacterial Resistance in Ureaplasma Species and Mycoplasma hominis Isolates from Urine Cultures in College-Aged Females. *Antimicrob Agents Chemother*. **61** (2017). 10.1128/AAC.01104-17 PMC5610494 28827422

[25] Pereyre S, Renaudin H, Charron A, Bebear C, Bebear CM (2006). Emergence of a 23S rRNA mutation in Mycoplasma hominis associated with a loss of the intrinsic resistance to erythromycin and azithromycin. J Antimicrob Chemother..

